# Supplementary material for: CL-Informer: Long time series prediction model based on continuous wavelet transform
Source: PLoS One. 2024 Sep 13;19(9):e0303990. doi: 10.1371/journal.pone.0303990 (PMC11398660; doi:10.1371/journal.pone.0303990)
Supplement: Attachment — Submitted filename: Response to Reviewers.pdf [file pone.0303990.s007.pdf]

List of Responses

Dear Editor Aamna Mohammed AlShehhi, PhD and Reviewers：

Thank you for your letter and for the reviewers’ comments concerning our manuscript entitled “CL-Informer: Long Time Series Prediction Model Based on Continuous Wavelet Transform” (ID:PONE-D-24-03744). Those comments are all valuable and very helpful for revising and improving our paper, as well as the important guiding significance to our researches. We have studied comments carefully and have made correction which we hope meet with approval. Revised portion are marked in red in the paper. The main corrections in the paper and the responds to the reviewer’s comments are as flowing:

Responds to the reviewer’s comments:

Reviewer #1:

1.Response to comment: （Can the authors explain why they used these five time series datasets in this paper? So can CL-Informer handle bigger data?）

Response:Thank you very much for your professional review of our articles. The use of these 5 data sets is firstly to reflect the model's generalization in multiple fields. Secondly, ETTh1 and ETTh2 in ETT data adopt the same time granularity to verify whether the prediction effect of the model will be improved under the same type of data. ETTm1 verifies that time series prediction with different granularity in the same domain will also improve, while ECL and Wheather data verify the model's generalization in different domains. Larger data sets can be handled.

2.Response to comment: Why in S2_Table includes ETTh2, but in S1_Table doesn’t?

Response:Thank you very much for your question. ETTh2 is not included in S2_Table because ETTh1 and ETTh2 are in the same domain, with the same time granularity and the same type of data, so we did not make multivariate prediction for ETTh2 in multivariate prediction.

3.Response to comment: In Figure 3, the x-axis is the prediction length in dataset ETTh2, which is {24, 48, 168, 336, 720}, but in the main manuscript content, it says the ETTh2 data set with input length of 168 and prediction length of {24, 48, 96, 168, 720}, which one is correct?

Response:We are very sorry for our miswriting, but should have followed the predicted length of the picture as {24, 48, 168, 336, 720}. (Amended page 6, paragraph 2, line 7.)

4.Response to comment: From Fig 4, the authors claimed as the support length of the Gaus wavelet increases, the prediction performance gradually improves. For example, MSE from 0.06 (gaus1) to 0.05 (gaus8) is only 0.01 improved, it seems like not a strongly improved.

Response:We agree with the reviewer's opinion on this issue, but in fact, the MSE of

CL-Informer gaus1 is 0.06445934, and the MSE of Gaus6 is 0.04488082, which has an increase of nearly 0.02. Moreover, each experiment was performed ten times, and the average MSE of the ten experiments of Gaus6 was about 0.02 higher than that of the ten experiments of Gaus1 wavelet. (We have redrawn Figure 4 to Figure 5 on page 7.)

5.Response to comment: Line 6 in paragraph one of the ‘Results and analysis’ part needs to be modified, that sentence is not finished.

Response:We are very sorry for our careless mistake. Thank you for reminding me. We rewrote the unfinished part of the sixth line of "Results and analysis." (The changes are made in the second paragraph of "Results and analysis" on page 11, and lines 3 to 7 have been modified)

6.Response to comment:（Some figures can be merged as one figure, such as Figure 7 and Figure 8, the same datasets, but one is for MSE and the other is for MAE score, which can be put into one figure. The authors need to pay attention that in some figures they used MSE, but in others, they used MSE score.）

Response:We have corrected it according to the reviewer's comments. We merged Figure 7 and Figure 8 into Figure 8 and changed the 'MSE' and 'MSE score' in the legend to MSE. (Revised as Figure 8 on page 11)

7.Response to comment: It’s better to add some legends for the figures, like Figure 9, to make it more readable for the readers.

Response:We have corrected according to the reviewer's comments. We added a legend to Figure 9, adding a legend illustration of the X and y coordinates. (Revised as Figure 9, page 13).

8.Response to comment: It’s better to let a native speaker go through this whole manuscript and modify the grammar issues.

Response:Thanks for your suggestion. We have tried our best to polish the language in the revised manuscript.

Special thanks to you for your good comments.

Reviewer #2:

1.Response to comment: Quality of Fig 4,5,7, need improved

Response:As suggested by the reviewer, we have redrawn Figure 4 and modified the quality of Figure 5 and Figure 7. (Modified as Figure 5 on page 7, Figure 6 on page 8, and Figure 8 on page 11)

2.Response to comment: In the abstract and conclusion, it is necessary to introduce the superiority of CL-Informer's performance in a quantified manner.

Response:Considering the reviewers' suggestions, we quantitatively introduced the advantages of CL-Informer in the abstract and conclusion. (Changes are made to the 9th

to last line of the summary on page 1 and the second paragraph of the conclusion on page 15.)

3.Response to comment: It is necessary to compare with other state-of-the-art algorithms to demonstrate the performance of the proposed method.

Response:We appreciate the reviewer's suggestion and compare MSE and MAE with other algorithms in Table 1 and Table 2.

4.Response to comment: Each variable in the formula needs to be explained.

Response:Thank you for your careful examination. We will add variables that are not explained in the formula.

5.Response to comment: page 5/8, before and ater Fig 3, redundant description.

Response:We apologize for our carelessness. In our resubmitted manuscript, the description of redundancy has been revised. Thank you for your correction.(The redundant description in Figure 4 (original Figure 3) has been deleted on page 6.))

6. Response to comment: Each image needs to be explained, clarifying what the symbols in the pictures represent.

Response:Thanks for your suggestion; we will add an explanation of the symbols in the picture.

7.Response to comment: The discussion section needs to include a quantitative analysis of the superiority of the proposed method and discuss which strategies have improved the performance of the algorithm.

Response:Thanks for your suggestion; we have added a quantitative analysis of the advantages of the proposed method in the discussion section (revised on page 14, lines 2 to 5).

8.Response to comment:（The abstract and conclusion sections need improvement.）

Response:We think this is an excellent suggestion. We introduced the advantages of CL-Informer by adding quantitative methods to the abstract and conclusion. (Changes are made to the 9th to last line of the summary on page 1 and the second paragraph of the conclusion on page 15.)

Special thanks to you for your good comments.

Reviewer #3:

1.Response to comment： The embedding layer based on wavelet transform is designed, it is obviou that the frequency division operation is carried out first, after the wavelet transform operation, how the data are input to the embedding layer of the model, please explain.

Response:We think this is a good suggestion. We added Figure 3 for the architecture

diagram of the CWT embed layer and rewrote the CWT embed layer. (The changes are in Figure 3 on page 5 and the last paragraph on page 5)

2.Response to comment:Thank you for your advice. "Distillation operation" In this article, we removed the "distillation" operation from the Informer model and added the LSTM layer. Please indicate whether replacing the "distillation" operation of the Informer model with LSTM makes sense.

Response:Thank you for your advice. "Distillation operation" is deleted because although it improves the model's generalization ability, it will affect LSTM's extraction of features that are long dependent on time series during distillation operation. We once used a CL-Informer model that did not remove the "distillation" operation, and its MSE, MAE, showed a low MAE value for a CL-Informer model that did not remove the "distillation" operation. (Modified at lines 5-8, paragraph 2, page 2)

3.Response to comment: There is no specific formula for positional embedding in the embedding layer, please explain in detail the specific operation of the embedding layer.

Response:Thanks to your suggestion, we have rewritten the CWT embedding layer (changes are made in the last paragraph of page 5).

4.Response to comment: Modify Figure 2, which does not clearly reflect the overall structure of the layer and the operation process.

Response:Thanks for your suggestion; we have added the architecture diagram of the embedded layer in Figure 3-CWT to supplement the overall structure of the layer in Figure 2. (Revised as Figure 3 on page 5)

5.Response to comment: The introduction of the CL-informer model proposed in the paper is not clear enough.

Response:Thanks for your suggestions; we will try our best to improve our presentation of the model.

Special thanks to you for your good comments.

Reviewer #4:

1.Response to comment: in general, most equations do not flow with the text. I.e.

Response:Thanks to your suggestion, we have modified the fluidity of the equation.

2.Response to comment: Many datasets (ETTh2, ETTm1) are discussed in the wavelet selection part of the paper before being described. All datasets should be described before being used.

Response:We have made corrections based on the reviewer's comments. 2. Data ETTh1 and ETTm1 are described before wavelet selection. (Amended at page 6, paragraph 1, lines 3 to 7)2.Response to comment: Each image needs to be explained,

clarifying what the symbols in the pictures represent.

3.Response to comment: Questions about wavelet selection and insert S1 as supplementary information.

Response:Thanks to the reviewer's suggestion, we slightly adjusted the part of the wavelet selection. We first added the architecture diagram of the CWT embedding layer in Figure 3 and rewrote the CWT embedding layer. Then, the choice of wavelet will be introduced to make the article smoother. (The changes are in Figure 3 on page 5 and the last paragraph on page 5)

4.Response to comment:"CNN" and "The approximate method used in Nyströmformer may not apply to longer sequences "are not defined.

Response:We have made corrections based on the reviewer's comments. We have a brief mention of CNN and a rewrite of the pros and cons of Nystromformer's long series predictions. (Changes are made on lines 1 to 2 and lines 22 to 24 of page 2)

5.Response to comment: Problem definition - "The determination of whether to use single-element or multi-element prediction depends on the value of dy ⩾ 1". Remove, this was defined above the Eq.

Response:Thanks to the reviewer's suggestion, the multi-element prediction was removed depending on the value of dy⩾1. (Modify the last paragraph of the Problem definition on the second page)

6.Response to comment: Formula (5) is not necessary because all are included in the formula (4), and an equation is added that describes "by summing over Wt along the second element."

Response:Thanks to the reviewer's suggestion, we deleted the original formula 5, added a new formula 5,6, and added the variable description. (Modified by Formula 5 on page 5 and Formula six on page 6)

7.Response to comment: The number of wavelets is infinite, so the sentence does not make sense: "Due to the extensive experimental workload, not all wavelets are based" on comparison.

Response:Thanks to the reviewer's suggestion, we deleted the section and rewrote it. (Amended first paragraph, page 15)

Special thanks to you for your good comments.

Other changes:

1. We made a structural change to the "Results and Analysis" section, bringing the original first paragraph under Figure 8. (Amended on page 11)
2. We changed the position of Table 1 under "Datum line" and put it into the "Cell time prediction:" of "Result and Analysis" to ensure a smoother article.(Revised at page 16)
3. We divide the first paragraph of "Results and Analysis" into two paragraphs.

(Revised at page 15, paragraphs 1 and 2)

4. We are sorry for our carelessness. We have corrected the Informer model in Table 3 with MSE of 288 input length and 720 output length, and MAE of 576 input length and 1440 output length to be the same as the experimental data. (The Informer's input length is 288 with an output length of 720 MSE, and the MAE with an input length of 576 and an output length of 1440 has been corrected in Table 3 on page 14.)

We tried our best to improve the manuscript and made some changes. These changes will not influence the content and framework of the paper. Moreover, we did not list the changes here but marked them in red in the revised paper.

We appreciate the Editors/Reviewers' warm work earnestly and hope the corrections will be approved.

Once again, thank you very much for your comments and suggestions.
